# Supplementary material for: Risk factors for multidrug-resistant and carbapenem-resistant Klebsiella pneumoniae bloodstream infections in Shanghai: A five-year retrospective cohort study
Source: PLoS One. 2025 May 22;20(5):e0324925. doi: 10.1371/journal.pone.0324925 (PMC12097643; doi:10.1371/journal.pone.0324925)
Supplement: S1 Table — (DOCX) [file pone.0324925.s001.docx]

**S1 Table. Definitions of each variable included in risk factors analysis for multidrug-resistant *Klebsiella pneumoniae* and carbapenem-resistant *Klebsiella pneumoniae* bloodstream infections**

| Variables | Definition |
| --- | --- |
| Age, years | Age at *Klebsiella pneumoniae* BSI diagnosis |
| Gender, male | As described in section “Gender” of the medical records |
| Smoking | As described in section “Smoking” of the medical records |
| Alcohol drinking | As described in section “Alcohol use” of the medical records |
| Combination therapy | Usage of antibiotics (more than one type of antibiotic at the same time) in the prior 90 days before blood collection date of the first positive culture |
| Comorbidities | *According to International Classification of Diseases-10th Revision codes (ICD-10) [1]* |
| Chemotherapy or radiotherapy | Receiving chemotherapy or radiotherapy treatment as described in medical records |
| Malignancy | C00-C80, C7A, C81-C96, D00-D09 |
| Disease of the circulatory system | I00-I99 |
| Hypertension | I10-I16 |
| Cerebrovascular disease | I67 |
| Heart failure | I1, I3, I50 |
| IHD | I25 |
| Endocrine, nutritional, and metabolic diseases | E00-E89 |
| Diabetes mellitus | Including Type 1 and Type 2 |
| Respiratory diseases | J00-J99 |
| Diseases of the genitourinary system | N00-N99 |
| Diseases of the gastrointestinal system | K00-K99 |
| Healthcare exposure | *In the prior 90 days before blood collection date of the first positive culture* |
| Time at risk, days | Interval between hospital admission and blood sample collection date of the first positive culture |
| Length of hospital stay, days | Total days of hospital stay |
| ICU stay | Stayed in ICU |
| Length of ICU stay, days | Total days of ICU stay |
| Invasive procedures | *In the prior 90 days before blood collection date of the first positive culture* |
| Surgery | As described in section “Surgery” of the medical records |
| Paracentesis | Including thoracentesis, lumbar puncture, bone marrow aspiration, abdominal paracentesis |
| Invasive ventilation | Invasive ventilation |
| Indwelling catheterization | Insertion of indwelling devices, including AC, CVC, urinary catheter, gastric tube, drainage tube |
| AC | Insertion of arterial catheter |
| Days of AC | Total days of indwelling arterial catheter |
| CVC | Insertion of central venous catheter |
| Days of CVC | Total days of indwelling central venous catheter |
| Urinary catheter | Insertion of urinary catheter |
| Days of indwelling urinary catheter | Total days of indwelling urinary catheter |
| Gastric tube | Insertion of gastric tube |
| Days of indwelling gastric tube | Total days of indwelling gastric tube |
| Drainage tube | Insertion of drainage tube |
| Days of indwelling drainage tube | Total days of indwelling drainage tube |
| Drug usage | *Any drug treatment in the prior 90 days before blood collection date of the first positive culture, including corticosteroids, immunosuppressor and antibiotics* |
| Corticosteroids | Administration of corticosteroids |
| Immunosuppressor | Administration of immunosuppressor, including cyclophosphamide, methotrexate, cyclosporine, tacrolimus |
| Antibiotics | Any antibiotic usage |
| Glycopeptides | Administration of any glycopeptide |
| Quantity, DDD | Total quantity of all the glycopeptides used |
| Vancomycin | Administration of vancomycin |
| Quantity, DDD | Total quantity of vancomycin |
| Linezolid | Administration of linezolid |
| Quantity, DDD | Total quantity of linezolid |
| Aminoglycosides | Administration of any aminoglycoside |
| Quantity, DDD | Total quantity of all the aminoglycosides used |
| Carbapenems | Administration of any carbapenem |
| Quantity, DDD | Total quantity of all the carbapenems used |
| Imipenem | Administration of imipenem |
| Quantity, DDD | Total quantity of imipenem |
| Meropenem | Administration of meropenem |
| Quantity, DDD | Total quantity of meropenem |
| Cephalosporins | Administration of any cephalosporin |
| Quantity, DDD | Total quantity of all the cephalosporins used |
| β-lactam/β-lactamase inhibitor combinations | Administration of any β-lactam/β-lactamase inhibitor combination |
| Quantity, DDD | Total quantity of all the β-lactam/β-lactamase inhibitor combinations used |
| Piperacillin-tazobactam | Administration of piperacillin-tazobactam |
| Quantity, DDD | Total quantity of piperacillin-tazobactam |
| Cefoperazone-sulbactam | Administration of cefoperazone-sulbactam |
| Quantity, DDD | Total quantity of cefoperazone-sulbactam |
| Fluoroquinolones | Administration of fluoroquinolones |
| Quantity, DDD | Total quantity of fluoroquinolones |

BSIs, bloodstream infections; IHD, ischemic heart disease; ICU, intensive care unit; AC, arterial catheter; CVC, central venous catheter; DDD, defined daily dose.

References:

[1] Centers for Disease Control and Prevention. *International Classification of Diseases-10th Revision codes*. 2019. Available from: <https://www.cdc.gov/nchs/icd/icd-10-cm.htm> (last accessed September 2024)
