# Supplementary material for: Risk factors for multidrug-resistant and carbapenem-resistant Klebsiella pneumoniae bloodstream infections in Shanghai: A five-year retrospective cohort study
Source: PLoS One. 2025 May 22;20(5):e0324925. doi: 10.1371/journal.pone.0324925 (PMC12097643; doi:10.1371/journal.pone.0324925)
Supplement: Attachment — Submitted filename: Response_to_Reviewers_auresp_2.docx [file pone.0324925.s005.docx]

Dear reviewer,

Thank you very much for your valuable comments and suggestions. We have responded to your comment and revised the manuscript accordingly. We highlighted all the revisions throughout the manuscript in yellow. Please see our responses in the below.

If the data set for this write-up has been used in previous publication (s), in order to conform to ethical standards, this must be clearly stated in the methodology section and the new or entirely different research question this write-up seeks to address unambiguously stated as well.

**Response:** Thank you. We have added the statement “We investigated incidence, antimicrobial resistance, crude 30-day mortality rate, and risk factors for crude 30-day mortality of KP BSIs using the same dataset of this manuscript (S1 File) and published the findings in 2024 [5]. In this study, we investigated temporal distribution of proportion of KP BSIs attributed deaths, department distribution of KP BSIs and risk factors for MDRKP BSIs and CRKP BSIs which are entirely different research questions. There is no overlap in research content of the two studies.” in the methodology section as suggested, please see lines 82-88.
